# Effect of different sous-vide cooking temperature-time combinations on the functional and sensory properties of goose meat

**DOI:** 10.1016/j.psj.2024.103701

**Published:** 2024-04-01

**Authors:** M. Wereńska

**Affiliations:** Department of Food Technology and Nutrition, Wroclaw University of Economics and Business, Wroclaw, 53-345, Poland

**Keywords:** goose meat, sous-vide, functional properties, sensory evaluation

## Abstract

The effect of goose meat sous-vide (**SV**) cooking at 6 combinations of temperature (60°C, 80°C) and time (4, 6, 12h) on selected functional properties was investigated. The study conducted an assessment of cooking loss (**CL**), moisture content, pH, longitudinal (**LS**), and transverse (**TS**) shrinkage, shear force (**SF**), texture profile analysis (**TPA**), color parameters (L*, a*, b*, C, h°), ΔE and carried out sensory evaluation. A total of 168 breast muscles (BM with and without skin) from 17-wk-old “Polish oat geese” were utilized. The CL was affected by both cooking temperature and time. The CL for meat with skin was higher than for without ones, and it was lower for both kinds of meat cooked at 60°C than at 80°C for all cooking times. The LS was higher than the TS. The higher shrinkage was stated for meat cooked at 80°C. There was a reduction in moisture content and slightly increasing pH by increasing temperature and prolonging cooking time. For both kinds of meat, the highest moisture retention was stated at 60°C/4h, and the lowest in samples heated at 80°C/12h. The samples cooked at 60°C were characterized by a higher L* value than those at 80°C. The a* values were higher for samples cooked at 60°C than those at 80°C, whereas b* were higher for meat cooked at 80°C. The SF exhibited a trend of lower values at 60°C compared to samples cooked at 80°C and it increased with prolonged cooking time. The value of hardness, cohesiveness, springiness, gumminess, and chewiness for meat cooked at 60°C increased, and for samples cooked at 80°C decreased with increasing cooking time. It was no significant differences in sensory scores for overall palatability for both kinds of meat cooked at 60°C and 80°C. Goose meat cooked at different time and temperature combinations showed extremely desirable overall palatability. Taking into account all discussed parameters, the optimal combination seems to be 60°C/4h.

## INTRODUCTION

Recently, as a result of technological development, the lifestyle of people around the world has changed. Consumers are attentive to sustaining healthy lifestyles; therefore, there is a great possibility to influence their perspectives, attitudes, and purchase intentions toward processed food. Demand is increasing for products high-quality, easy to prepare, and freshly, not sterilized, but have extended shelf life and have fewer synthetic additives and preservatives. This phenomenon has been noticed by the food industry, thus the producers have focused on accelerated food preparation and presentation techniques, half-ready and ready-to-eat foods. Cooked meats are an important segment of the meat industry to be used either in ready-meals, as a delicatessen product, or as an ingredient in meat-based food products. Hence, the meat processors in the meat industry also have been motivated by the meat consumers toward healthy food processing. Consumer acceptance of meat is strongly influenced by the eating quality (Drummond and Sun, 2006; Costa et al., 2011; Grujić et al., 2014; Nowicka et al., 2018). The consumers choose a cooking method that produces high-quality meat products having a favorable texture and taste. The eating quality of meat is improved when applying heat treatments at low temperatures for a prolonged time (Christensen et al., 2013). These conditions are met in the sous-vide (**SV**) method. Sous-vide simply means “under vacuum” in French. It illustrates the process in which food is cooked in heat-stable vacuumed containers (plastic pouches) under mild controlled temperatures for specific time durations followed by low-temperature storage or may be directly served to the customers. The desired temperature-time combination for food preparation can be regulated by altering the temperature (53–81°C) and time (2–48h) in the water circulation of the water bath or by circulating heat (convection) and steam of convection steam ovens/combi steamer ovens (Baldwin, 2012; Roldán et al., 2013; Jeong et al., 2018). Both, temperature and cooking time have a large effect on the eating quality of meat cooked by sous-vide processing. Precise temperature and time control in this method preserves the technological, sensory, microbial, and nutritional properties of meat and meat products while having the potential to reduce the costs of materials, labor, and storage (Ayub and Ahmad, 2019). The temperature is critical during cooking because it causes changes mainly in the texture and color of the meat products. It is due to the transformation in the connective tissue proteins, sarcoplasmic, and myofibrillar proteins structure and in myoglobin redox status (Tornberg, 2005; Mancini and Hunt, 2005; Baldwin, 2012; Zielbauer et al., 2016). Heating at low temperatures reduces the cooking loss, compared to higher temperatures, which leads to a more juicy product (Vaudagna et al., 2002; Dall Aaslyng et al., 2003; Christensen et al., 2013). Nevertheless, particularly long heating time, up to 30 h, leads to less juicy but tenderer meat. Therefore, it is important to find a balance between these 2 parameters (temperature and cooking time) to meet consumers' needs (Mortensen et al., 2012; Christensen et al., 2013). It should be noted that it was made many studies with sous-vide cooking for beef, pork, lamb, chicken, and turkey meat, but there is a gap in the literature regarding the use of sous-vide technology for thermal processing of goose meat. The alternative to the most popular heat treatment methods for goose meat may be the sous-vide technique, as a result of which a product classified as “ready to eat” (**RTE**) is created. To improve the quality of goose meat, it is necessary to estimate the precise combination of cooking temperature and time of heat processing, as this combination is a pivotal factor that influences the functional and sensory properties of this meat. Therefore, this study evaluated the effect of sous-vide cooking (6 combinations of temperature × time) of goose breast meat and also effect of kind of meat x heat treatment on its functional properties and sensory quality.

## MATERIAL AND METHODS

### Meat Samples

The material for the study were boneless breasts (*Pectoralis major*) from White Kołuda® geese (“Polish oat geese”) which were slaughtered after 17 wk of life and delivered by the producer. The birds were reared on the same commercial farm and fed on the same completely concentrated diet (Wołoszyn et al., 2020). Before the slaughter, geese were subjected to feed withdrawal for 12 h. Next, the birds were slaughtered in a poultry plant, according to the EU regulations (Council Regulation EC No 1099/2009). The raw material was stored in refrigeration conditions (+4 ± 1°C, 24h), washed, trimmed, and calibrated to 25 to 27 mm of thickness. One serving portion of the breast with skin was 480 ± 10 grams and without skin 389 ± 35 grams. A total of 24 geese's breast muscles (12 samples with skin and 12 samples without skin) were used at each temperature × time. In addition, 24 breast muscles were used for performing analyses of raw meat. Any food additives were used in the experiment. Before heat treatment muscle mass, pH, color parameters L*, a*, b*, length and width of each muscle, and moisture content were analyzed in raw material.

### Sous-Vide Cooking Procedure

The design of the sous-vide treatments included combinations of 3 cooking times (4, 6, and 12 h) and 2 temperatures (60 and 80°C). This resulted in 6 treatments consisting of all combinations of time and temperature (60°C/4h - SV60/4; 60°C/6h - SV60/6; 60°C/12h - SV60/12; 80°C /4h - SV80/4; 80°C/6h – SV80/6; 80°C/12h - SV80/12). The experimental design was replicated twice. Before cooking, samples (with and without skin) were placed into vacuum polyamide/polyethylene bags (thickness of 90 μm, heat resistance (HR) of -40°C/+120°C, O_2_ permeability of 9 cm^3^/m^2^ per 24h at 4°C/80% HR and water steam permeability of 1.2 g/m^2^ per 24h) and further heat-sealed using vacuum sealing machine (Profi Line 40+, Hendi, Robakowo, Poland) with extent of vacuum 99.6%. Thereafter, the samples were cooked in thermostated water baths (model SW 22, Julabo GmbH, Seelbach, Germany) at different temperature-time combinations. The water baths have been preheated to 60 and 80°C (in independent experiments) and the samples were maintained within the water bath for 4, 6, and 12h for each temperature. The heating time of 4, 6, and 12h was counted from the moment of reaching the temperature of 60, and 80°C of the muscle core (the hand-held thermometer was used in an additional control sample - Thermometer, DT-34 with the probe, Termoprodukt, Bielawa, Poland). Once the cooking process had finished, the pouches were removed from the water bath and submerged in ice-cold water (2°C) for 1 h and next put in chilled storage at 4°C for 24h. Then, they were allowed to equilibrate to room temperature (21°C, 3h), and pH, cooking loss (**CL**), moisture content, longitudinal (**LS**) and transverse (**TS**) shrinkage, color parameters such as L*, a*, b*, chroma (**C**), hue angle (h°), and the individual differences (**ΔE**) in L*, a*, and b* values, shear force (**SF**), texture profile analysis (**TPA** - hardness, springiness, cohesiveness, gumminess, chewiness), and sensory evaluation (flavor and aroma typical for goose meat, tenderness, juiciness, cohesiveness, springiness, and overall palatability) were determined.

### Cooking Loss

To calculate the percentage of cooking loss, the raw muscles (with and without skin) were weighed, and then the bags with sous-vide samples (after cooled down to room temperature) were opened, and these samples were weighed. Cooking loss was calculated from differences in the weight before (Wb) and after sous-vide cooking (Wsv).

CL(%)=(Wb−Wsv)/Wb×100%

### Longitudinal and Transverse Shrinkage of the Whole Muscle

Longitudinal and transverse shrinkage was determined as the percent reduction in the cooked sample length along and across the muscle fiber, respectively, compared with the raw sample. The maximum lengths and widths of the raw and cooked samples were measured using a laser measuring tool (Flex ADM1 FLEX, Germany). The LS and transverse TS shrinkage were determined from differences in the length and width before (L_b_, T_b_ respectively) and after sous-vide cooking (L_sv_, T_sv_ respectively).

Shrinkages were calculated as follows:

LS(%)=(Lb−Lsv)/Lb×100%,andTS(%)=(Tb−Tsv)/Tb×100%

### Moisture Content

The moisture content of goose raw and sous-vide meat (with and without skin) was determined using AOAC methods (AOAC International, 2016). Before analysis, the breast muscles were ground separately (mesh size 2 mm) and homogenized with an IKA homogenizer (SBS-MR-2500, IKA-Werke GmbH & Co., Staufen, Germany). The moisture content (%) was calculated by weight loss of the samples (3 g) after oven-drying at 102°C (to constant weight - 12h) in a Memmert laboratory dryer (UN 75, Schwabach, Germany) (950.46B, p. 39.1.02).

### pH Measurement

The pH measurement in the muscle (with and without skin) was performed using a Double Pore Slim combination dagger electrode (Hamilton Robotics, Reno, NV) connected to a CyberScan pH 1500 pH meter (Eutech Instrument, Vernon Hills, IL). The measurement consisted of inserting an electrode into the muscle. The final result was the mean of 3 measurements taken in different locations in the muscle.

### Color Measurement

CIE Lab color space - lightness (L*), redness (a*), and yellowness (b*) was measured with a Minolta Colorimeter (CR-310, Konica Minolta Co. Ltd, Osaka, Japan) equipped with D65 standard illuminant, 8 mm viewing port and 10° standard observer before and after sous-vide cooking on a fresh cut. The assessments were carried out on 5 preselected locations at the surface of each breast muscle. The colorimeter was calibrated before each trial with a standard white reflectance calibration plate Y = 94.2; x = 0.313; y = 0.324. The device was adjusted to perform 5 internal measurements, automatically calculating the average value of L*, a*, b*, Chroma (C), hue angle (h°), and the individual differences (ΔE) in L*, a*, and b* values were calculated using the following equations (CIE, 1986):

$$C=\left(a^{*}2+b^{*}2\right)1/2$$

h° = tg^−1^ (b*/a*), were h° = 0° for reddish hue and h° = 90° for yellowish hue.

$$\Delta E={\left[{\left(\Delta L^{*}\right)}^{2}+{\left(\Delta a^{*}\right)}^{2}+{\left(\Delta b^{*}\right)}^{2}\right]}^{1/2}$$

Where: ΔL*, Δa*, Δb* is the difference between raw and sous-vide meat in lightness, in color in the red-green axis, and in color in the yellow-blue axis (respectively).

### Warner–Bratzler Shear Force and Texture Profile Analysis

Texture analysis of cooked meat (with and without skin) was based on SF and TPA using an Intron Universal Testing Machine (Model 5543; Instron Corp., Canton, Norwood, MA) with the Warner–Bratzler blade (V-shaped) operating at a crosshead speed of 50 mm/min. For analysis of SF, 3 cores from each muscle (2.54 cm diameter x 1 cm height) were removed parallel to the muscle fiber orientation, and the SF values were measured. The mean of the peak SF from 3 replicates from each experimental sample was used as an estimate of SF (expressed in N). The TPA was performed methods published by Bourne (1978, 2002). The 3 samples (2.54 cm diameter x 1.0 cm height) cut out from each cooked breast muscle were compressed to 70% of their original height at a speed of 5 mm/min and a wait time of 5 s in a 2-cycle compression test. Texture parameters for hardness, springiness, cohesiveness, gumminess, and chewiness were calculated using Bluehill 3-testing Software Instron.

### Sensory Evaluation

The sensory analysis of sous-vide cooked goose meat was a separate experiment, performed in independent sessions. A total of 144 sous-vide cooked goose breast samples (12 with skin and 12 without skin for each combination) were evaluated during 6 sessions. The sensory quality of sous-vide-cooked goose meat was conducted using a quantitative descriptive analysis (**QDA**) (Stone et al., 2020). A panel of nine judges was familiar with goose meat and descriptive analysis procedures. The team consisted of non-smokers (all females aged 34–53). The training process and evaluation of the trained panel adhered to the guidelines outlined in the standard ISO 8586:2012 “Sensory analysis - General guidelines for the selection, training, and monitoring of selected assessors and expert sensory assessors,” established by the International Organization for Standardization (**ISO**). Panelists underwent training specifically focused on assessing heat-treated meat raw materials. The sensory evaluation was carried out at the laboratory located in the Department of Food Technology and Nutrition in Wroclaw (Poland) with all requirements of the international standards EN-ISO 8589:2010. Selection of quality descriptors (Table 1, the definitions of sensory characteristics were the same as in previous Wołoszyn et al., 2020) was carried out by the ISO 13299:2016 procedure. The following 6 attributes were evaluated: flavor and aroma typical for goose meat, tenderness, juiciness, cohesiveness, springiness, and overall palatability. Samples (1.5 × 1.5 × 1.5 cm) were labeled with randomly assigned 3-digit codes and served to the judges on identical white glass plates. Unsalted crackers and distilled water were provided to clean and refresh the palate between samples. The samples were analyzed for the intensity of sensory descriptors. To determine the intensity of each sensory attribute, a 10-point scale expressed in conventional unit (**CU**) with specific word anchors was used. It was 2 repetitions from each muscle. The opinions expressed by each assessor were recorded on an evaluation sheet.

**Table 1:** Descriptors used in sensory evaluation and definitions of points scale (CU).

Score	Flavor and aroma typical for goose meat	Tenderness	Cohesiveness	Juiciness	Springiness (elasticity)	Overall palatability
0 - 2	absence (extremely low intensity)	extremely tough	extremely low (extremely easy to fragmentation)	extremely dry	no elasticity	unacceptable
3 - 4	Sufficiently	moderately though	Low	moderately dry	low elasticity	sufficient
5 - 6	pleasant-good	moderately tender	moderately low	moderately juicy	moderately elasticity	good
7 - 8	very pleasant	tender	High	juicy	elasticity	very good
9 - 10	excellent (extremely high intensity)	extremely tender	extremely high (extremely difficult to fragmentation)	very juicy	extremely elasticity	excellent (extremely desirable)

### Statistical Analysis

The data were analyzed as a completely randomized design using a 2-way ANOVA concerning the time of sous-vide cooking (4, 6, and 12 h) and temperature of sous-vide cooking (60°C, and 80°C) as a factorial design (3 × 2) (written in the table as a-f), according to the following linear model: Y_ij_ = μ + A_i_ + B_j_ + (AB)_ij_ + e_ij_, where Y_ij_ = value of trait (the dependent variable); μ = overall mean; A_j_ = effect of time of sous-vide cooking; B_j_ = effect of temperature of sous-vide cooking; (AB) = interaction, and e_ij_ = random observation error, using Statistica®13.3 software (Statsoft Inc., 2019). Moreover, it was made also 2-way ANOVA concerning the heat treatment (time x temperature) of sous-vide cooking and kind of meat (with and without skin) as a factorial design (6 × 2) (written in the tables as x-y), according to the following linear model: Y_ij_ = μ + A_i_ + B_j_ + (AB)_ij_ + e_ij_, where Y_ij_ = value of trait (the dependent variable); μ = overall mean; A_j_ = effect of heat treatment (time × temperature); B_j_ = effect of kind of meat (with skin and without skin); (AB) = interaction, and e_ij_ = random observation error, using Statistica 13.3 software (Statsoft Inc., 2019). The statistical significance of the differences between the averages of the groups was calculated using Tukey's test and was at a level of P≤0.05. The tables present the average values and their standard deviation.

## RESULTS AND DISCUSSION

### Cooking Loss, Temperature Shrinkage (LS and TS), Moisture Content, and pH

Cooking loss is an important factor to consider because it is directly related to juiciness which could influence the consumer's perception of the final product. The cooking loss is defined as total liquid and soluble matter lost from the meat during cooking and it is influenced by different factors such as the quality of the raw meat, genetics of the animals, and cooking conditions. This loss relies on the mass transfer process during heat treatment (Haghighi et al., 2021). The cooking loss from sous-vide cooked goose meat was affected by both cooking temperature (*P* = 0.001), cooking time (*P* = 0.001), and kind of meat x heat treatment (*P* = 0.001). There was also an interaction between cooking temperature and the time of heat treatment for both kinds of meat (*P* = 0.001). In general, the cooking loss increased at 60°C from 19.08 to 24.31% in meat without skin, and from 23.65 to 31.47% in samples with skin. At the temperature of 80°C, the cooking loss was from 31.66 to 36.74% in meat without skin, and from 42.18 to 43.98% with skin (Table 2). For meat with skin CL was higher than for without ones, because, an additional component loss during heat treatment for goose breast muscles with skin is subcutaneous fat. This difference can be attributed to the presence of subcutaneous fat in the skin layer of the goose breast muscles. During heat treatment, not only are the proteins denatured and water is lost, but the subcutaneous fat also undergoes changes. Subcutaneous fat has a relatively lower melting point compared to other components of the meat, such as muscle proteins. Therefore, during cooking, the subcutaneous fat melts and is rendered out from the meat along with the expelled fluids, contributing to a higher cooking loss in meat with skin (Xu et al., 2022). In the current study, the cooking loss in the SV meat samples cooked at 60°C was lower than at 80°C for all 3 cooking times. In meat without skin, in case SV60/4 the CL was significantly lower than for SV60/6 and SV60/12. There were no significant differences between SV60/6 and SV60/12 samples without skin. However, in the case of meat with skin, it was stated that a higher CL for SV60/12 than SV60/4 and SV60/6. There were no significant differences for SV60/4, and SV60/6 meat with skin. Although it was the highest CL for both kinds of meat SV80/12, there were no significant differences for SV80/4, SV80/6, and SV80/12. As expected, the cooking loss was higher as the cooking temperature increased and cooking time held for an extended period but the time had less influence than temperature. The CL is significantly related to the contraction of muscle fibers and connective tissue.

**Table 2:** Cooking loss (%), moisture content (%), pH (-), longitudinal and transverse shrinkage (%) of goose meat cooked sous-vide (n = 12 breast muscles with skin and n = 12 without skin for each kind of heat treatment).

Sample	Cooking time (T)	Parameters									
		Cooking loss (%)		Moisture content (%)		pH		Longitudinal shrinkage (LS) (%)		Transverse shrinkage (TS) (%)	
		without skin	with skin	without skin	with skin	without skin	with skin	without skin	with skin	without skin	with skin
Raw		-	-	73.11 ax ± 0.57	63.55^a^^y^ ± 0.36	5.72^bc^ ± 0.18	5.74^b^ ± 0.21	-	-	-	-
60° (SV60)	4h	19.08^c^^y^ ± 1.40	23.65^c^^x^ ± 2.28	68.99^b^^x^ ± 0.13	60.21^b^^y^ ± 0.25	5.81^bc^ ± 0.06	5.85^a^ ± 0.03	21.22^b^^x^ ± 1.68	17.80^c^^y^ ± 1.34	11.46^ab^^x^ ± 0.64	5.56^e^^y^ ± 0.01
	6h	23.84^b^ ± 1.40	24.65^c^ ± 2.30	67.71^bc^^x^ ± 0.12	60.11^b^^y^ ± 0.20	5.83^ab^ ± 0.03	5.79^b^ ± 0.05	21.40^b^^x^ ± 1.77	19.88^b^^y^ ± 0.98	12.15^a^^x^ ± 0.64	7.69^d^^y^ ± 2.15
	12h	24.31^b^^y^ ± 1.40	31.47^b^^x^ ± 2.42	67.62^c^^x^ ± 0.10	59.97^b^^y^ ± 0.17	5.90^a^^x^ ± 0.05	5.76^b^^y^ ± 0.04	20.41^b^^x^ ± 1.43	18.48^c^^y^ ± 1.22	10.86^b^ ± 2.34	10.81^c^ ± 2.35
80°C (SV80)	4h	31.66^a^^y^ ± 4.71	42.18^a^^x^ ± 1.30	62.36^d^^x^± 0.17	59.75^b^^y^ ± 0.07	5.76^c^ ± 0.05	5.74^b^ ± 0.03	31.01^a^ ± 3.51	32.15^a^ ± 3.84	12.43^a^^y^ ± 1.70	19.15^a^^x^ ± 2.69
	6h	34.21^a^^y^ ± 2.28	43.06^a^^x^ ± 3.62	62.27^d^^x^ ± 0.10	59.61^b^^y^ ± 0.24	5.75^c^ ± 0.05	5.79^b^ ± 0.05	31.73^a^ ± 2.64	33.48^a^ ± 2.11	12.58^a^^y^ ± 1.10	15.10^b^^x^ ± 2.89
	12h	36.74^a^^y^ ± 0.69	43.98^a^^x^ ± 2.30	60.40^e^^x^ ± 0.10	59.37^b^^y^ ± 0.13	5.82^bc^^x^ ± 0.05	5.76^b^^y^ ± 0.05	31.97^a^ ± 3.08	32.14^a^ ± 2.20	12.95^a^^y^ ± 1.78	17.26^ab^^x^ ± 3.69
Time (T)		0.001	0.001	0.001	0.844	0.001	0.001	0.001	0.004	0.014	0.001
Temperature (Tm)		0.001	0.001	0.001	0.244	0.001	0.001	0.017	0.001	0.508	0.001
Temperature x time (Tm × T)		0.001	0.001	0.019	0.991	0.762	0.062	0.108	0.105	0.233	0.001
Kind of meat x heat treatment		0.001		0.001		0.001		0.074		0.001	

a-e – Different letters in columns means statistically significant differences (*P* ≤ 0.05).

x-y – Different letters in rows means statistically significant differences (*P* ≤ 0.05). ± Standard deviation.

Meat shrinkage during cooking can be described as a 2-dimensional process. Transverse shrinkage, or shrinkage perpendicular to the muscle fiber direction, is reported to start between 35°C and 45°C. As meat temperature is increased from 50 to 60°C, there is a significant reduction in muscle fiber diameter. This decrease in diameter has been attributed to the reduction of the inter-fiber spacing due to myofibrillar protein denaturation and, after that, to the severe contraction of collagen fibers (parallel to the sarcomeres). At temperatures over 60°C, shrinkage proceeds, both in diameter and in the longitudinal axis. Longitudinal shrinkage, or shrinkage parallel to the direction of the muscle fibers, leading to either sarcomere length or fiber length change, is completed by 90°C (Offer et al., 1984; Palka and Daun, 1999; Tornberg, 2005; Dominguez-Hernandez et al., 2018; Vaskoska et al., 2020). Transverse shrinkage is attributed to expanding the gap space between muscle fibers and endomysium, thereby reducing the amount of drip generation. Longitudinal shrinkage causes substantial water loss which increases with temperature (Baldwin, 2012). Alternately, the denaturation of connective tissue protein which is responsible for the longitudinal shrinkage of muscle accounted for most of the cooking loss of meat, mainly due to the shortening of muscle fibers (Tornberg, 2005). Increasing holding time has a, relatively, smaller effect than increased temperature on diameter changes (Dominguez-Hernandez et al., 2018). During sous-vide cooking the goose samples showed both kinds of shrinkage but transverse shrinkage occurred to a lesser extent. The longitudinal shrinkage was affected by cooking temperature (*P* = 0.017 and *P* = 0.001), cooking time (*P* = 0.001 and *P* = 0.004) for both kinds of meat, but LS was not affected by kind of meat x heat treatment (*P* = 0.074). The transverse shrinkage was affected by cooking temperature for meat with skin (*P* = 0.001), time of heat treatment for both kinds of meat (*P* = 0.014 and *P* = 0.001) and also TS was affected by kind of meat x heat treatment (*P* = 0.001). There was also an interaction between cooking temperature and the time of heat treatment (*P* = 0.001) for meat with skin. The longitudinal shrinkage was higher than the transverse ones (Table 2). The longitudinal shrinkage at 60°C was higher for meat without skin than with skin, but for 80°C there were no significant differences. In case of meat with skin transverse shrinkage was significantly lower at 60°C than at 80°C, while for meat without skin, there were no significant differences between SV60/4, SV60/6, and SV80/4, SV80/6, SV80/12. Generally, the higher shrinkage was stated for meat cooked at 80°C. Compared to 60°C, the higher amount of CL for cooked meat at 80°C was probably due to the higher shrinkage of muscle fibers. Whereas, Becker et al. (2016), observed that the long time-low temperature (**LT–LT**) (53°C/20h, 58°C/20h) pork samples showed a markedly transverse shrinkage (11.2–13.8%) while longitudinal was significantly smaller (4.0–7.1%). They stated higher cooking loss for pork LT-LT 58°C/20h compared to LT–LT 53°C/20h samples. Christensen et al. (2010) stated that, both longitudinal and transverse shrinkage were significantly more pronounced at the LT–LT 58°C compared to the LT–LT 53°C samples and accompanied by a higher cooking loss. Results presented for sous-vide goose meat, concerning CL were consistent with the data obtained by other authors for different materials. Also, Becker et al. (2015) confirmed the results of Christensen et al. (2011), that temperature was the main factor affecting cooking loss at low temperatures (53, and 58°C), and long time (10, 20, and 30h) treated pork meat while holding time had a smaller impact. In an experiment of Kurp et al. (2022) with pork meat subjected to sous-vide with different combinations of temperature (60, 65, 70, 75°C) and time (1, 1.5, 2, 3, 4h) it was observed the cooking loss was in the range from 18.16 to 36.66%, and it increased significantly with temperature and cooking time. The interaction of temperature and cooking time also showed a significant effect on cooking loss. These authors stated that cooking losses tended to be higher when higher temperatures were applied. However, longer cooking time at 50°C resulted in lower cooking loss, while at higher temperatures the cooking time effect was not significant. The study of Jeong et al. (2018) showed that when the cooking temperature increased from 61 to 71°C the weight loss of pork meat was higher but there was no impact of time cooking (45 and 90 min.). The authors stated that the amount of cooking loss was influenced by temperature to a greater extent than by the cooking time. On the other hand, Rezler et al. 2023 stated that in pork meat subjected to sous-vide, the CL increased within the temperature range of 60 to 85°C, but it slightly increased along with the cooking time from 6 to 18h. Hwang et al. (2019) established that the CL in pork meat tended to increase along with temperature (50, 55, and 60°C), but longer cooking times (12 and 24h) at 50°C resulted in a smaller loss. When the cooking temperature was higher than 55°C, the cooking time did not affect cooking loss. However, after cooking at 60°C for 24h the cooking loss was usually higher than after 12h. When meat is subjected to heat during cooking, the muscle fibers and connective tissue undergo structural changes due to the denaturation of proteins. This denaturation causes the muscle fibers to contract, leading to the expulsion of moisture and soluble substances from the meat. Additionally, the connective tissue, which surrounds and supports the muscle fibers, also undergoes changes, contributing to the overall loss of moisture during the cooking process. Therefore, the degree of cooking loss is significantly influenced by the extent of contraction experienced by the muscle fibers and connective tissue as a result of protein denaturation. García-Segovia et al. (2007) show that as the temperature raised from 60 to 80°C and the time from 15 to 60 min, cooking loss of beef meat increased from 10 to 38%. Głuchowski et al. (2020) reported that in chicken meat cooked at 64°C/60 min. the CL was smaller than at 66°C/80 min. and 75°C/35 min. Haghighi et al. (2021) stated that the CL of sous-vide chicken breast was affected by both cooking temperature (60, 70, 80, and 100°C) and cooking time (60, 90, 120, and 150 min.) and it was increased from 10.23 to 28.08%. In the study of Karpińska-Tymoszczyk et al. (2020), the cooking loss was higher in chicken meat processed at longer times (320 vs. 260 min., 200 vs. 140 min., 150 vs. 90 min.), and the greatest was observed in meat processed at the highest temperature (61 vs. 58°C, and 55°C). They concluded that the above could be attributed to water retention by collagen and advanced changes in protein structure under prolonged exposure to heat. Collagen, a major component of connective tissue in meat, has the ability to retain water, which contributes to the juiciness and succulence of the final cooked product. Moreover, as meat is subjected to extended cooking times, the proteins undergo further structural modifications, such as denaturation and cross-linking, which can affect their water-binding capacity and overall texture. Therefore, the combination of collagen's water-retaining properties and alterations in protein structure due to prolonged heating likely influences the cooking loss and texture of the cooked meat (Wang et al., 2022). Conventional heat treatment methods (oven roasting, boiling, pan frying, stewing, grilling, microwave cooking,) are characterized by higher cooking loss from goose meat (34.2–52.2%) (Wołoszyn et al., 2020; Wereńska, 2023) compared to the sous-vide method used in this work in a combination of three times and 2 temperatures (19.1–43.9%). The goose breast muscles without skin cooked sous-vide at 70°C/4h characterized a similar value of cooking loss (22.1%) to samples SV60, whereas CL from meat with skin reached a value of 32.2% and was similar to SV60/12 sample (with skin) (Wereńska, 2023). This is known, that moisture content is one of the important physicochemical characteristics in meat which plays a basic role in the palatability of meat. The moisture content in raw goose meat without and with skin was 73.11% and 63.55% respectively, and as a result of cooking it declined in both kinds of meat. The moisture content in cooked goose meat without skin ranged from 68.99 to 60.40% and in meat with skin from 60.21 to 59.37% depending on temperature, and time cooking (Table 2). The significant effect of temperature (*P* = 0.001), and time (*P* = 0.001) of cooking on the moisture content was noted for meat without skin and the interaction of these parameters was significant (*P* = 0.019) but, in the case of meat with skin was no effect of temperature and time of cooking (0.244–0.844). Moreover, it was noted the significant effect on kind of meat x heat treatment (*P* = 0.001). Meat without skin had a higher moisture content than samples with skin. Both kinds of meat cooked at 60°C for 4h showed the highest moisture retention, and the lowest moisture retention was observed in samples cooked at 80°C for 12h. There was a reduction in moisture content by increasing temperature and prolonging cooking time, too, but it was no significant differences in moisture content of meat with skin for all 6 combinations of temperature, and cooking time. Exposure of the meat samples to high temperatures leads to the denaturation of myofibrillar proteins and changes in the water-holding capacity of meat. Loss of water in cooked meat is caused by leakage of cellular juice under the influence of elevated temperature. Myofibrillar proteins shrink (it was explained above) and lose water during cooking. Additionally, a contraction of the perimysial connective tissue seems to take place causing a compression of the muscle fiber bundles, which in turn encourages water to be released from the meat cut (Sánchez Del Pulgar et al., 2012, Roldán et al., 2013). Karpińska-Tymoszczyk et al. (2020) established that in all chicken samples subjected to sous-vide (55°C/260, and 320 min.; 58°C/140, and 200 min.; 61°C/90, and 150 min.) the water content significantly decreased, as a result of heat treatment. The authors stated that the above could be attributed to water retention by collagen whose hydrolysis begins at around 50°C and proceeds rapidly at 55 to 65°C. The moisture content of meat decreased significantly at 61°C and 58°C, when processing time was longer. In an experiment of Głuchowski et al. (2020) chicken meat cooked at higher temperatures (66°C/80 min., and 75°C/35 min.) (SV66 and SV75 respectively), had a lower water content than at 64°C/60 min (SV64). According to Haghighi et al. (2021), there was a reduction in moisture content in chicken meat by increasing the temperature from 60 to 80°C. Increasing cooking time from 60 to 150 min. at higher temperatures (70, and 80°C) caused a reduction in moisture content. In a study of Roldán et al., (2013) lamb loins cooked sous-vide at 80°C show lower moisture content than those cooked at 60°C and 70°C. Samples cooked at 60°C for 24h were characterized by lower water content than those cooked for 6h, but the magnitude of the observed changes was not correlated with heating time. Generally, the moisture content was affected by cooking temperature, but the time of cooking did not have a significant effect on moisture content. Jeong et al. (2018) reported that the sous-vide pork samples cooked at 71°C showed significantly lower moisture content than those cooked at 61°C. Both moisture content and cooking loss were affected by temperature. Similarly, in an experiment of Sánchez Del Pulgar et al. (2012) sous-vide pork meat cooked at 80°C showed significantly higher weight losses and lower moisture content than those cooked at 60°C. Not only temperature but the cooking time also had a significant effect on these 2 parameters. In the work of Kurp et al. (2022), the moisture content in raw pork meat was 72.45%, and as a result of cooking (60, and 65°C/2, 3, 4h, also 70, and 75°C/1, 1.5, 2h), it declined significantly to 62.32-68.91%. Meat cooked at 60°C for 2h showed the highest moisture retention. The significant effect of both temperature and time of cooking on the moisture content was noted, while the interaction of these parameters was not significant. In a study of Supaphon et al. (2020) the beef meat cooked sous-vide was characterized by much higher water loss at temperatures 70°C, and 80°C than 60°C. After 2h of cooking, samples cooked at 70°C lost more than twice as much water as the samples cooked at 60°C. Compared to 60°C, the large decrease in water holding capacity for sous-vide cooked meat at 70°C and 80°C was probably due to the shrinkage of muscle fibers because of connective tissue contraction and perhaps also because of new cross-linkages in the coagulated myofibrillar system as described by Roldán et al. (2013).

The concentration of hydrogen ions (**pH**) is an important determinant of meat quality. The pH value of meat influences its water-holding capacity, and the reaction pathways of the Maillard reaction, and therefore affects the palatability and storage stability of cooked meat (Karpińska-Tymoszczyk et al., 2020; Kurp et al., 2022). The pH test results reveal no big differences between raw (5.72 for meat without skin, and 5.74 for meat with skin) and cooked goose meat without (5.75–5.90) and with skin (5.74–5.85) in thermal conditions 60, and 80°C/4, 6, 12h. In general, the pH of meat subjected to sous-vide at different combination temperatures and time of cooking increased slightly. The significant effect of both temperature (*P* = 0.001) and time (*P* = 0.001) of cooking on the pH of meat was noted, while the interaction of these parameters was not significant (*P* = 0.762 and *P* = 0.062). Moreover, it was noted the significant effect on kind of meat x heat treatment (*P* = 0.001). According to Fletcher et al. (2000) and Becker et al. (2016), an increasing temperature might cause an increase in pH mainly due to the protein denaturation and the change in protein charge. Faustman et al. (2023) reported that the increase in pH of cooked meat may be due to a loss of acidic amino group and Vasanthi et al. (2007) exposure of basic amino residues and/or a formation of free hydrogen sulfide. Similarly, Bıyıklı et al. (2020) observed that increasing the cooking temperature from 65 to 75°C and the cooking time from 20 to 60 min. caused a slight increase in the pH of the sous-vide turkey cutlet. Although the temperature-time combinations did not affect the pH values, the individual effects of the temperature and time parameters were found to be significant. In turn, Haghighi et al. (2021) observed that the pH value of sous-vide chicken breast fillets cooked at different temperatures and time combinations slightly increased (and ranged from 6.07 to 6.30). The pH was affected by temperature and the interaction between temperature and time. In an experiment of Hwang et al. (2019), the pH of pork meat sous-vide was affected by cooking conditions. All samples (50, 55, and 60°C/12, 24h), showed similar pH to raw pork meat. Also, Głuchowski et al. (2020) described that chicken breasts prepared at 64°C/60 min. (SV64) had the least change in pH (6.16) compared to raw (6.14) material. The samples SV66 and SV75 were characterized by higher pH values (6.30, 6.32 respectively). Lower protein denaturation caused the lower pH values of pork cooked at 53°C/20h than at 58°C/20h (Becker et al., 2016). In the study of Kurp et al. (2022) analysis of pH values did not reveal significant differences between raw (5.78) and cooked pork meat (5.80–5.91) in thermal conditions: 60, 65, 70, and 75°C/1, 1.5, 2, 3, and 4h. Park et al. (2020) stated that in cooked chicken meat there was no significant difference in pH within the SV60 or SV70 treatments for 1, 2, and 3h.

### Color Parameters

Changes in meat color during heating can result from various chemical and physical processes such as protein denaturation, oxidation, the Maillard reaction (enzymatic browning), and non-enzymatic browning. The ultimate color depends on the extent of ferrihemochrome formation, which in turn is a product of the initial proportionality of the myoglobin, and the final concentration of undenatured oxyMb or deoxyMb (King and Whyte, 2006; Ling et al., 2015; Guo et al., 2017). All instrumental color parameters for sous-vide goose meat (Table 3) were significantly affected by the temperature of cooking (*P* = 0.001–0.033) and L*, a*, and h° were additionally significantly affected by the time of cooking. The analysis revealed differences in lightness L* (42.80–59.51) for the different times in different temperatures. It was significant differences in L* value between SV60/4, and SV80/4h; SV60/6, and SV80/6; SV60/12, and SV80/12. It was no significant differences in L* value between SV60/4 and SV60/6, and between SV80/4 and SV80/6. In both temperatures, the L* value was lower for 12h time compared to the remaining samples. The samples cooked at 60°C were characterized by a higher L* value than those at 80°C. According to Sánchez del Pulgar et al. (2012) and Rees et al. (2003), this result may be related to the greater amount of exuded water, which remains impregnating the water's surface. This occurrence has been pointed out as a cause of higher L* values. The Lab values were measured on a fresh cut of cooked goose meat. In this regard, it has been observed that those samples that retained more water during cooking, released some water to the surface during the slicing process before color measurement. In contrast, the samples that lost a greater amount of water during cooking seemed to have a smaller amount of exuded water on the surface, giving it a dry aspect (Sánchez del Pulgar et al., 2012). Moreover, all sous-vide cooked meat samples, compared to raw meat, become brighter due to the increased reflectance and scattering of light by denatured and aggregated sarcoplasmic and myofibrillar proteins (which was manifested by an increase in the L* value) (Young and West, 2001; Christensen et al., 2011). Also, thermal treatment contributes to the denaturation of myoglobin, resulting in the formation of metmyoglobin in meat. Metmyoglobin denatures further upon heating, resulting in a darker brown color. Heat treatment with high temperature, in addition to denaturation, usually causes the globin molecule to unfold, resulting in the formation of globin-hemichrome or ferrihemochrome, known as the dull brown pigment formed upon heating (Zhang and Wang, 2012; Roldán et al., 2013; Suman and Joseph, 2013). There are various discussions in the literature regarding changes in the L* value of sous-vide cooked meat when the temperature increases. Some authors describe any significant differences in L* values (Rinaldi et al., 2014; Becker et al., 2015, 2016; Haghighi et al., 2021; Karpińska-Tymoszczyk et al., 2020; Kurp et al., 2022), some of them lower L* values (Sánchez del Pulgar et al., 2012; Roldán et al., 2013; Ismail et al., 2019a; Zhang et al., 2022), while others describe higher L* values (García-Segovia et al., 2007; Christensen et al., 2011; Becker et al. 2016; Ismail et al., 2019b; Park et al., 2020). The presented results of the L* value for sous-vide goose meat are in accordance with data detected by Sánchez del Pulgar et al. (2012) for pork, Roldán et al. (2013) for lamb, Zhang et al. (2022) for duck meat. These authors deduced that all measured color parameters were affected by cooking temperature, too. Thus, sous-vide pork, lamb, and duck meat cooked at 60°C showed higher L* values than those cooked at 70°C, or 80°C. In this study for goose meat, redness (a*) for all cooked samples was significantly lower, and yellowness (b*) was significantly higher, than for raw ones. Moreover, it was noted that the value of a* for meat cooked at 60°C (4 and 6h) was significantly higher than at 80°C (4, 6, and 12h). It was previously stated that the intensity of the a* parameter of cooked meat is inversely related to the degree of denatured myoglobin. Such process takes place between 55°C and 65°C and continues till 75°C or 80°C (King and Whyte, 2006; Roldán et al., 2013; Karpińska-Tymoszczyk et al., 2020). Accordingly, the a* goose meat parameters were significantly affected by cooking temperature (*P* = 0.033) and by cooking time (*P* = 0.001), showing a more intense red color (higher a* values) samples cooked at 60°C than those cooked at 80°C. These results indicate higher myoglobin degradation as the cooking temperature increases. Whereas b* parameters were higher for sous-vide goose meat cooked 4, 6, and 12h at 80°C, compared to cooked 4 and 6h at 60°C. There was a significant increase in b* values as a consequence of cooking temperature (*P* = 0.001). It was most likely due to the formation of metmyoglobin and further heat-denaturation of this protein, giving rise to a brownish color. There is a controversial discussion in the literature concerning a* and b* value changes during heating. Some authors describe any changes in a* values (García-Segovia et al., 2007; Karpińska-Tymoszczyk et al., 2020) and b* values (Sánchez del Pulgar et al., 2012; Rinaldi et al., 2014; Park et al., 2020) at higher temperatures for sous-vide cooking. Some of them describe lower a* values (Sánchez del Pulgar et al., 2012; Becker et al., 2016, Ismail et al., 2019a,b; Zhang et al., 2022; Haghighi et al., 2021), and lower b* values (García-Segovia et al., 2007), while others describe higher a* values (Rinaldi et al., 2014; Park et al., 2020) and b* values (Ismail et al., 2019a,b; Zhang et al., 2022) at higher temperatures for sous-vide cooking. The presented results of a* value for sous-vide goose meat are in line with data stated by Sánchez del Pulgar et al. (2012) for pork meat, Roldán et al. (2013) for lamb loin, and Zhang et al. (2022) for duck meat cooked at 60, and 80°C. However, the data concerning b* value of sous vide goose meat are consistent with the results: of Zhang et al. (2022) for duck; Ismail et al. (2019b), for beef cooked at 45, 65°C/3h, and Ismail et al. (2019a) for beef cooked at 60, 65, 70, 75°C/6,12h. The cooking changed the hue angle (h^o^), and Chroma (C) parameters of goose meat, too (Table 3). Chroma, that is, the saturation of the meat's color relates to the concentration of myoglobin and its degree of denaturation. This attribute is more predominant with higher concentrations of myoglobin and at a lower rate of denatured myoglobin (Ledward, 1992). Sous-vide cooking caused a reduction in the C parameter compared to raw meat (23.48 vs. 17.85–20.34). The slightly lower C value for sous-vide cooked samples at 80°C than at 60°C and significantly lower compared to raw meat showed that they were brighter (less distant to the L* axis of the CIE Lab system). In all cooking samples value of the h° parameter was significantly higher (28.88–36.72) than raw meat (6.35). This means that the color of these samples was more yellow and was closer to the b* axis in the CIE color space. The hue angle is the result of the chemical state of myoglobin and its value is inversely proportional to the a* value. Thus, goose samples of sous-vide cooked at 60°C showed lower h° values, most likely due to a lower rate of denatured myoglobin than those cooked at 80°C. Similarly, Sánchez del Pulgar et al. (2012) stated that the C values for pork samples SV60 were higher than those SV80, while the samples SV60 showed lower h° compared to SV80. In the study of Rinaldi et al. (2014) chroma beef samples SV75 showed a lower value compared to SV100 although not significantly. Finally, the hue angle resulted higher in SV75 compared to SV100 and inversely related to a* values. The goose results for C and (h°), are in line with data obtained by Shin et al. (2023) for duck meat subjected to sous-vide cooking. The hue angle for duck increased, while the C decreased with increasing the temperature and cooking time (from 50 to 80°C and 1h to 3h). In Table 4 there were presented the values of the ΔE parameter. The delta E parameter can be used to assess changes in meat color before and after various processes, such as sous-vide processing, storage, or additives to meat products. Delta E is typically used in the food industry to monitor and maintain the color uniformity of meat products. It may be deduced that the color of the cooked samples changed in comparison to raw meat. The value of ΔE for raw and heat treatment samples ranged between 12.93 (SV80/12) and 23.42 (SV60/4). The calculated values of the ΔE parameter showing the differences in color between raw meat and the tested sous-vide goose meat were as follows: SV60/4 > SV60/6 > SV60/12 > SV80/4 > SV80/6 > SV80/12. The lowest difference of the ΔE parameter (0.82) was calculated in SV80/4 & SV80/6 pair samples and according to (Třešňák, 1999) indicated minute or perceptible color differences. The highest difference of the ΔE parameter (17.11) was established between SV60/4 & SV80/12 samples and it showed visible color differences. According to the CIE Lab classification, unnoticeable color is when the parameter ΔE has reached a value lower than 2. This value is in this study for pairs SV60/4 & SV60/6 and for SV80/4 & SV80/6. The remaining samples had ΔE parameters higher than 2, which means that these pairs had visible differences. García-Segovia et al. (2007) observed ΔE values in the range from 13 to 15 for sous-vide cooking beef cooked up 15 to 60 min. They concluded that observing the variation range in Chroma, hue, and ΔE at different temperatures, times, and treatments there were not very important. In the work of Jeong et al. (2018) the value of ΔE was the highest for pork meat SV61/45 min. (12.22 NBS - National Bureau of Standards) and lowest for SV 71/90 min. (7.19 NBS). These values were defined as very large and large differences, respectively. The cooking temperature exerts strong effects, but cooking has limited influence on color attributes.

**Table 3:** CIE lab – color parameters of goose meat cooked sous-vide (n = 48 breast muscles - raw meat, n = 24 - cooked breast muscles for each kind of heat treatment).

Thermal treatment	Cooking time	L*	a*	b*	C		h°
Raw	-	37.78^e^ ± 2.62	22.56^a^ ± 2.02	2.43^c^ ± 0.29	23.48^a^ ± 1.60		6.35^d^ ± 0.85
60° (SV60)	4h	59.51^a^ ± 3.10	17.79^b^ ± 1.89	9.74^b^ ± 1.15	20.34^b^ ± 1.52	28.88^c^ ± 4.55	
	6h	58.17^a^ ± 2.06	17.01^bc^ ± 2.76	9.96^b^ ± 0.83	19.77^bc^ ± 2.45	30.80^bc^ ± 4.44	
	12h	55.84^b^ ± 3.47	15.78^cd^ ± 2.17	10.65^ab^ ± 0.81	19.06^bc^ ± 2.08	34.25^ab^ ± 3.06	
80°C (SV80)	4h	46.08^c^ ± 2.44	14.93^d^± 1.60	11.07^a^ ± 0.84	18.62^bc^ ± 1.45	36.70^a^ ± 3.44	
	6h	45.27^c^ ± 3.17	14.78^d^ ± 1.42	11.04^a^ ± 1.23	18.47^bc^ ± 1.65	36.72^a^ ± 2.79	
	12h	42.80^d^ ± 3.74	14.33^d^± 1.49	11.05^a^ ± 0.74	17.85^c^ ± 1.70	36.51^a^ ± 3.00	
Time (T)		0.001	0.001	0.112	0.142		0.001
Temperature (Tm)		0.001	0.033	0.001	0.001		0.001
Temperature x time (Tm × T)		0.365	0.402	0.113	0.693		0.027

a-e - Different letters in columns means statistically significant differences (*P* ≤ 0.05); lightness (L*), redness (a*), and yellowness (b*), as well as the calculated hue angle (h^°^) and Chroma (C); ± standard deviation.

**Table 4:** Color differences (∆E) in sous-vide cooked goose meat.

Parameters	Raw meat (R)	Temperature/Time					
		60°C/4h (SV60/4)	60°C/6h (SV60/6)	60°C/12h (SV60/12)	80°C/4h (SV80/4)	80°C/6h (SV80/6)	80°C/12h (SV80/12)
Raw meat (R)	x	23.42	22.43	20.97	14.20	13.81	12.93
60°C/4h (SV60/4)	23.42	X	1.57	4.28	13.80	14.61	17.11
60°C/6h (SV60/6)	22.43	1.57	X	2.72	12.32	13.14	15.64
60°C/12h (SV60/12)	20.97	4.28	2.72	x	9.81	10.62	13.13
80°C/4h (SV80/4)	14.20	13.80	12.32	9.81	x	0.82	3.33
80°C/6h (SV80/6)	13.81	14.61	13.14	10.62	0.82	x	2.51
80°C/12h (SV80/12)	12.93	17.11	15.64	13.13	3.33	2.51	x

### Warner–Brazler Shear Force and Texture Profile Analysis

The SF is an important eating quality character due to its impact on texture and consumer acceptance. While SF is a good measure of the initial tenderness, TPA gives more detailed information on texture properties (Jeong et al., 2018). The values for SF and the different textural parameters (including the TPA analysis) are shown in Table 5. For both kinds of meat, the temperature and time of sous-vide cooking affected the SF value (*P* = 0.001). Also, kind of meat x heat treatment affected the SF value (*P* = 0.001). It was for SF (*P* = 0.007 and *P* = 0.049) and all TPA parameters (*P* = 0.001–0.003) interaction between time × temperature, too. The temperature affected TPA parameters, for both types of muscles, such as hardness, gumminess, and chewiness (*P* = 0.001). On the other hand, there was no significant difference in cohesiveness, and springiness value for meat with skin (*P* = 0.241; *P* = 0.111 respectively). For both kinds of meat, the time affected hardness (*P* = 0.001), cohesiveness (*P* = 0.001, *P* = 0.050) springiness (*P* = 0.001, *P* = 0.045), and chewiness (*P* = 0.049). Taking account, the correlation between kind of meat, and heat treatment, only hardness (*P* = 0.024) and springiness (*P* = 0.015) had significant values for both parameters. Shear force for both types of goose meat cooked at 80°C was higher for 6h, than for 12h, but the difference was not significant. It exhibited a general trend of lower SF values when cooked at 60°C compared to samples cooked at 80°C. Additionally, SF values for all samples increased with prolonged cooking time. Probably these results were caused by the denaturation of myosin (55–60°C), actin (≈80°C), and collagen contraction (56–65°C) (Baldwin, 2012). The denaturation of myofibrillar proteins leads to meat toughening (Vaskoska et al., 2020). Tornberg (2005) suggests that the contraction of the connective tissue, mainly occurring after 65°C gives rise to an increase in the elasticity of the meat by forming a much denser material in the temperature region of 65 to 80°C and thereby a tougher meat. The toughness increased at temperatures higher than 60°C up to 80°C because the breaking strength of muscle fibers increased. Many researchers (Christensen et al., 2011; Roldán et al., 2013; Rinaldi et al., 2014; Becker et al., 2016; Dominguez-Hernandez et al., 2018; Jeong et al., 2018; Ismail et al., 2019a; Naqvi et al., 2021) have confirmed that despite the temperature, duration of cooking also affects the tenderness of the meat. The value of hardness, cohesiveness, springiness, gumminess, and chewiness generally for goose samples cooked at 60°C increased, and for samples cooked at 80°C decreased with increasing cooking time. Significant lower hardness values (105.05 and 105.80N) for both types of goose samples cooked at 80°C for 12h compared to 6h (137.66 and 128.43N) and 4h (163.32 and 149.10N) according to Baldwin (2012) could be caused by an extensive disintegration of the perimysium around muscle bundles. This was not detected in the SF test (Table 5) since in this case, the measured force is that needed to transversely cut the meat fibers, while the hardness parameter in the TPA refers to the force to compress the sample to 70% of its height (described in Material and Methods), which is affected by the binding force between bundles. Moreover, the measurement of cohesiveness (Table 5) in the TPA test indicated such generation of a fragile structure as a consequence of long cooking time at a higher temperature, since samples cooked at 80°C for 12h were significantly less cohesiveness (0.41, and 0.42) than those from any other combinations of time and temperature. The hardness (except SV60/12), springiness, gumminess, cohesiveness (except SV80/12), and chewiness parameters were lower in meat cooked at 60°C than those at 80°C. In this study, the significant (*P* < 0.05) positive correlation coefficients for the pairs SF-CL, SF-hardness, and CL-hardness were calculated and they fall within the range of moderate values (0.4–0.7 data not shown in the table). Under these conditions of temperature and heating time, where CL was lower, SF and hardness were also lower. It was stated that among all goose samples cooked at 6 temperature and time combinations, the best texture profile had SV60/4 meat. Similar to the present work Palka and Daun (1999); Ismail et al. (2019a,b); Jeong et al. (2018), Biyikli et al. (2020); Haghighi et al. (2021) observed that SF value for beef, pork, turkey, and chicken meat was affected by cooking temperature and it was increased by increasing temperature. They explained these results based on myofibrillar proteins (myosin and actin) denaturation and connective tissue proteins (mainly collagen) changes and concluded also that this result might be associated with higher moisture content and lower cooking loss of samples cooked at lower temperatures. Cooking at low temperatures reduces the protein-protein association and gelation and increases water. Kurp et al. (2022) stated no significant differences in SF value, and hardness for pork loin cooked at 60 to 65°C for 2, 3, and 4h. Moreover, at higher temperatures (70–75°C), the SF value decreased and the hardness increased with increasing cooking time (1, 1.5, 2h). Also, Park et al. (2020) presented no significant differences in the hardness and chewiness of chicken breast at 60°C for 1, 2, and 3h. It was no significant differences in cohesiveness, springiness, and gumminess at 60°C and 70°C for all times, but hardness and chewiness significantly increased at 70°C for 1, 2, and 3h. Christensen et al. (2011) stated that prolonged time (from 3 to 20h) did not caused higher SF value at any temperatures. Vaudagna et al. (2002); García-Segovia et al. (2007); Christensen et al. (2011); Sánchez Del Pulgar et al. (2012); Becker et al. (2016); and Naqvi et al. (2021) reported a decrease in SF values for beef, and pork meat as the temperature was raised (generally from 50 to 80°C). On the other hand, Ismail et al. (2019a) stated a decrease in SF values for beef muscles as the sous-vide time increased. With increasing cooking time García-Segovia et al. (2007) noted a decrease in toughness, Naqvi et al. (2021) in hardness, chewiness, and cohesiveness, Sánchez Del Pulgar et al. (2012) in hardness, springiness, cohesiveness, chewiness. In the study of Roldán et al. (2013), there was a trend to a decrease, both in hardness, and in SF values with cooking time at any given cooking temperature (60, 70, 80°C). This could be due to greater collagen solubilization and the gel formation with longer cooking time, while myofibrillar shrinkage would have reached its maximum even with the shortest cooking time and thus, it would have no further increased with longer times. Prolonged cooking time stronger affected the tenderness values than the cooking temperature. The results stated by many authors for various types of meat and different sous-vide conditions varied significantly. Roldán et al. (2013) stated the cause of the changes in texture parameters as a consequence of temperature cooking and time is very difficult to elucidate because in many experiments different temperature and time conditions of the sous-vide process were used. However, it is known that the thermohydrolysis of connective tissue (mainly collagen), and changes in sarcoplasmic and myofibrillar proteins occur at different temperature and time of thermal treatment. Both the intramuscular connective tissue and the myofibrillar component contribute to toughness. In many cuts, connective tissue is the major source of toughness, but the myofibrillar component is sometimes dominant and referred to as actomyosin toughness (Baldwin, 2012).

**Table 5:** SF and TPA parameters of goose meat cooked sous-vide (n = 12 breast muscles with skin and n = 12 without skin for each kind of heat treatment).

Thermal treatment	Cooking time	SF (N)		Hardness (N)		Cohesiveness		Springiness		Gumminess (N)		Chewiness (N)	
		without skin	with skin	without skin	with skin	without skin	with skin	without skin	with skin	without skin	with skin	without skin	with skin
60° (SV60)	4h	65.86^d^^x^ ± 5.57	55.70^c^^y^ ± 4.09	52.14^d^ ± 4.09	62.71^d^ ± 6.19	0.48^c^ ± 0.02	0.45^ab^ ± 0.05	0.51^cd^ ± 0.01	0.50^ab^ ± 0.06	25.02^e^ ± 4.51	28.33^d^ ± 5.46	12.72^d^ ± 2.62	13.89^d^ ± 2.14
	6h	81.66^c^^x^ ± 4.38	75.56^b^^y^ ± 4.21	57.78^d^ ± 8.83	63.60^d^ ± 6.02	0.54^a^ ± 0.03	0.50^a^ ± 0.05	0.62^a^ ± 0.03	0.54^a^ ± 0.02	31.18^d^ ± 4.95	31.70^d^ ± 4.06	19.32^c^ ± 2.24	17.21^c^ ± 3.98
	12h	94.94^bc^^x^ ± 3.97	78.22^b^^y^ ± 5.46	110.90^c^ ± 5.41	111.52^bc^ ± 6.93	0.55^a^ ± 0.01	0.52^a^ ± 0.03	0.58^ab^ ± 0.03	0.55^a^ ± 0.03	60.96^b^ ± 3.19	58.36^b^ ± 5.27	35.47^b^ ± 3.83	32.11^a^ ± 3.59
80°C (SV80)	4h	84.04^c^ ± 7.57	81.32^b^ ± 3.78	163.32^a^^x^ ± 12.25	149.10^a^^y^ ± 11.93	0.54^a^ ± 0.04	0.50^a^ ± 0.05	0.56^bc^ ± 0.03	0.52^ab^ ± 0.04	88.70^a^^x^ ± 9.90	74.10^a^^y^ ± 5.70	49.52^a^^x^ ± 7.55	38.47^a^^y^ ± 6.29
	6h	115.19^a^^x^ ± 9.28	96.02^a^^y^ ± 9.33	137.66^b^ ± 8.41	128.43^b^ ± 11.18	0.50^b^ ± 0.03	0.52^a^ ± 0.07	0.53^c^ ± 0.03	0.54^a^ ± 0.03	68.84^b^ ± 4.32	66.39^ab^ ± 11.78	36.25^b^ ± 3.71	36.08^a^ ± 7.87
	12h	104.27^ab^ ± 7.75	103.33^a^ ± 8.86	105.05^c^ ± 8.13	105.80^c^ ± 10.07	0.41^d^ ± 0.03	0.42^b^ ± 0.04	0.47^cd^ ± 0.04	0.47^b^ ± 0.02	42.49^cd^ ± 3.77	44.27^c^ ± 3.85	20.20^c^ ± 2.96	20.91^b^ ± 2.03
Time (T)		0.001	0.001	0.001	0.001	0.001	0.050	0.001	0.045	0.081	0.086	0.049	0.049
Temperature (Tm)		0.001	0.001	0.001	0.001	0.001	0.241	0.001	0.111	0.001	0.001	0.001	0.001
Temperature x time (Tm × T)		0.007	0.049	0.001	0.001	0.001	0.001	0.001	0.003	0.001	0.001	0.001	0.001
Kind of meat x heat treatment		0.017		0.024		0.071		0.015		0.068		0.052	

a-e - Different letters in columns means statistically significant differences (*P* ≤ 0.05).

x-y - -Different letters in rows means statistically significant differences (*P* ≤ 0.05). ± Standard deviation.

In this experiment, it was obvious that the sous-vide cooking at 60°C for 4h had an advantage in producing tenderer goose meat compared to remaining cooking conditions.

### Sensory Evaluation

The sensory evaluation of goose meat cooked sous-vide is presented in Table 6. The time of cooking affected descriptors such as tenderness (for meat with and without skin), and juiciness (for meat with skin) (*P* = 0.001–0.044). The kind of meat and heat treatment affected sensory evaluation parameters (flavor and aroma typical for goose meat, tenderness, and juiciness) (*P* = 0.001–0.023). The temperature of cooking influenced only juiciness (for meat with skin). There was interaction T × Tm only for juiciness. No notable distinctions in sensory scores were observed between meat prepared at 60°C and 80°C for 4, 6, and 12h in terms of flavor and aroma typical for goose meat, cohesiveness, springiness, and overall palatability. The higher scores for all sensory descriptors were established for meat with skin compared to samples without skin, moreover, it was no significant differences in overall palatability for both kinds of meat. According to the scores provided by the panelists (as described in Table 1), the goose meat was characterized by extremely high flavor and aroma intensity. The texture was noted as extremely tender and the meat was described as very juicy, too. Therefore, in general, goose meat (both types) subjected to sous-vide cooking in 6 different time and temperature combinations showed extremely desirable overall palatability. Karpińska-Tymoszczyk et al. (2020) stated that there were no significant differences in aroma, tenderness, taste, and overall acceptance for sous-vide chicken breast fillets cooked at 58°C (140 and 200 min) and at 61°C (90 and 150 min). Only juiciness at 61°C decreased with the increase of time of cooking. Moreover, at 55°C (260 and 320 min) these authors observed that aroma increases and tenderness decreases with the increasing time of cooking. Biyikli et al. (2020) stated no significant differences in taste, flavor, chewiness, and toughness for turkey cutlets cooked at 65, 70, and at 75°C for 20, 40, and 60 min. These authors show only differences in juiciness, which decreased with increasing cooking time (from 20 to 60 min) at 65°C, and at 75°C. Soletska and Krasota (2015) used boiling in water bath (at 95°C, 30 min) and SV (at 63, 65°C/60 min) technology for chicken breast muscles. The sensory evaluation shows that sous-vide samples had more impressive taste and smell, firm and juicy texture, and good consistency (note 5 for all parameters on a 5-point scale) than those cooked in a water bath (note ≤ 4). According to Park et al. (2020), the scores for flavor (at 60, and 70°C) and overall acceptability (at 60°C) of sous-vide cooked chicken breast increased, whereas the juiciness, tenderness, and chewiness decreased with increasing time of cooking (from 1 to 3h). On the other hand, Becker et al. (2016) stated that juiciness decreased, and tenderness of pork meat had no significant differences with increasing temperature (from 53 to 58°C) of LT-LT cooking. The meat was perceived as being very tender. Polak and Markowska (2019) stated that turkey breast meat, regardless of the time and temperature of sous-vide cooking was characterized by similar sensory scores from 4 to 5 points, for taste, smell, and color parameters. Although the texturometric analysis did not show any statistically significant differences in the hardness of the samples, noticeable changes were confirmed organoleptically. Meat prepared at temperature of 64°C for 120 min was rated the highest, with a score of over 5, and that prepared at 64°C for 60 min received the lowest score (3.5). Whereas, Kurp et al. (2022) observed no differences in overall appearance, aroma intensity, tenderness, juiciness, flavor acceptability, and overall palatability of pork loin subjected to sous-vide cooking at 60°C and 65°C for 2, 3, and 4h, as well as at 70°C and 75°C for 1, 1.5, and 2h. Moreover, the authors stated no interaction between temperature and time for aroma, tenderness, juiciness, flavor acceptability, and overall palatability. The time had an impact on tenderness, juiciness, and overall acceptability, and the temperature on tenderness, juiciness flavor acceptability, and overall palatability.

**Table 6:** Sensory evaluation of goose meat cooked sous-vide (CU) (n = 12 breast muscles with skin and n = 12 without skin for each kind of heat treatment).

Thermal treatment	Cooking time	Flavor and aroma typical for goose meat		Tenderness		Juiciness		Cohesiveness		Springiness (elasticity)		Overall palatability	
		without skin	with skin	without skin	with skin	without skin	with skin	without skin	with skin	without skin	with skin	without skin	with skin
60° (SV60)	4h	8.38^y^ ± 0.23	9.56^x^ ± 0.42	9.00^bc^ ± 0.46	9.13^b^ ± 0.35	8.75^b^^y^ ± 0.27	9.25^ab^^x^ ± 0.54	8.88^y^ ± 0.52	9.38^x^ ± 0.52	8.38^y^ ± 0.44	9.38^x^ ± 0.52	9.31 ± 0.46	9.44 ± 0.50
	6h	8.44^y^ ± 0.32	9.63^x^ ± 0.44	9.56^ab^ ± 0.18	9.25^b^ ± 0.18	9.00^ab^^y^ ± 0.53	9.75^ab^^x^ ± 0.27	9.00 ± 0.46	9.50 ± 0.53	8.63 ± 0.69	9.31 ± 0.46	9.31 ± 0.46	9.44 ± 0.42
	12h	8.44^y^ ± 0.32	9.69^x^ ± 0.37	9.88^a^ ± 0.23	10.00^a^ ± 0.00	9.25^ab^^y^ ± 0.46	9.81^a^^x^ ± 0.37	9.06^y^ ± 0.56	9.62^x^ ± 0.52	8.69^y^ ± 0.46	9.75^x^ ± 0.46	9.63 ± 0.52	9.81 ± 0.37
80°C (SV80)	4h	8.50^y^ ± 0.46	9.69^x^ ± 0.37	8.81^c^^y^ ± 0.65	9.63^b^^x^ ± 0.35	9.31^ab^ ± 0.37	9.63^ab^ ± 0.35	9.25 ± 0.38	9.50 ± 0.53	8.88^y^ ± 0.64	9.72^x^ ± 0.26	9.43 ± 0.42	9.63 ± 0.44
	6h	8.56^y^ ± 0.32	9.75^x^ ± 0.38	8.81^c^^y^ ± 0.46	9.81^b^^x^ ± 0.26	9.56^a^ ± 0.49	9.81^a^ ± 0.26	9.25 ± 0.53	9.56 ± 0.50	8.81^y^ ± 0.75	9.63^x^ ± 0.52	9.56 ± 0.50	9.50 ± 0.46
	12h	8.63^y^ ± 0.23	9.81^x^ ± 0.26	8.63^c^^y^ ± 0.44	9.44^b^^x^ ± 0.49	9.12^ab^ ± 0.23	9.19^b^ ± 0.53	9.31 ± 0.65	9.69 ± 0.46	8.88^y^ ± 0.58	9.69^x^ ± 0.46	9.50 ± 0.46	9.56 ± 0.50
Time (T)		0.700	0.645	0.044	0.001	0.233	0.043	0.700	0.700	1.000	1.000	1.000	1.000
Temperature (Tm)		0.135	0.256	1.000	1.000	0.006	0.597	1.000	1.000	1.000	1.000	1.000	1.000
Temperature x time (Tm × T)		0.944	1.000	0.051	0.204	0.031	0.003	1.000	1.000	1.000	1.000	1.000	1.000
Kind of meat x heat treatment		0.001		0.021		0.023		0.453		0.210		0.995	

a-c - Different letters in columns means statistically significant differences (*P* ≤ 0.05).

x-y - Different letters in rows means statistically significant differences (*P* ≤ 0.05). ± Standard deviation.

## CONCLUSIONS

Analyzing the results of the functional properties of goose meat processed sous vide in 6 variants of temperature and heating time, and also kind of meat (with and without skin) in 2 variants in 6 heat treatment it cannot be clearly stated which combination is the most suitable for this meat. Taking into account, cooking loss, moisture content, longitudinal and transverse muscle shrinkage, the optimal combination seems to be 60°C/4h. Generally, goose meat cooked at 60°C compared to 80°C showed a higher value of the L*, a* parameters, lower of b*, and SF and had more favorable texture parameters for the consumer. Sensory evaluation of goose meat demonstrated that it was characterized by an extremely desirable overall palatability, and it should be noted that the overall assessment was similar and statistically insignificant for muscles with and without skin. However, cutting force, cooking loss, moisture and longitudinal and transverse shrinkage were significantly different for muscles with and without skin. Carrying out further research on the nutritional value of goose meat and its microbiological stability is necessary to precisely determine the sous-vide processing conditions under which the meat will be of the highest quality.

## DISCLOSURES

The authors declare no conflicts of interest.
